# Studies of host preferences of wild-caught *Phlebotomus orientalis* and *Ph. papatasi* vectors of leishmaniasis in Sudan

**DOI:** 10.1371/journal.pone.0236253

**Published:** 2020-07-21

**Authors:** Arwa Elaagip, Ayman Ahmed, Michael David Wilson, Daniel A. Boakye, Muzamil Mahdi Abdel Hamid

**Affiliations:** 1 Department of Parasitology and Medical Entomology, Faculty of Medical Laboratory Sciences, University of Khartoum, Khartoum, Sudan; 2 Department of Parasitology and Medical Entomology, Institute of Endemic Diseases, University of Khartoum, Khartoum, Sudan; 3 Department of Parasitology, Noguchi Memorial Institute for Medical Research, University of Ghana, Accra, Ghana; 4 The END Fund, New York, NY, United States of America; Academic Medical Centre, NETHERLANDS

## Abstract

**Introduction:**

Understanding the feeding behavior and host choice of sand flies provides valuable information on vector-host relationships and elucidates the epidemiological patterns of leishmaniasis transmission. Blood meal analysis studies are essential for estimating the efficiency of pathogen transmission, assessing the relative human disease risk, and assist in identifying the other potential hosts of leishmaniasis. In Sudan and most of East Africa, there are large remaining gaps in knowledge regarding the feeding habits of phlebotomine vectors. The study aimed to identify the blood meal sources and, therefore, the host preferences of the principal vectors *Phlebotomus orientalis* and *Ph*. *papatasi* in leishmaniasis endemic areas of eastern and central Sudan.

**Materials and methods:**

Sand flies were collected from two endemic villages in eastern and central Sudan using CDC light traps and sticky traps. The phlebotomine sand flies were morphologically and then molecularly identified. The source of blood meal of the engorged females was determined using a multiplex PCR methodology and specific primers of cytochrome *b* gene of mitochondrial DNA for human, goat, cow, and dog. The detection of the *Leishmania* parasite was done using PCR.

**Results:**

The total number of collected female phlebotomine sand flies was 180. Morphological identification revealed the abundance of *Ph*. *orientalis* 103 (57.2%), *Ph*. *papatasi* 42 (23.3%), *Ph*. *bergeroti* 31 (17.2%), *Ph*. *rodhaini* 2 (1.1%) and *Ph*. *duboscqi* 2 (1.1%) in the study sites. Out of the 180 collected, 31 (17%) were blood-fed flies. Three species were blood-fed and molecularly identified: *Ph*. *papatasi* (N = 7, 22.6%), *Ph*. *bergeroti* (N = 9, 26%), and *Ph*. *orientalis* (N = 15, 48.4%). Blood meal analysis revealed human DNA in two *Ph*. *orientalis* (6.4%), hence, the anthropophilic index was 13.3%.

**Conclusions:**

Multiplex PCR protocol described here allowed the identification of blood meal sources of many vertebrate species simultaneously. The results indicate that wild-caught *Ph*. *orientalis* are anthropophilic in the study areas. Further studies on larger blood-fed sample size are required to validate the potential applications of this technique in designing, monitoring and evaluating control programs, particularly in investigating the potential non-human hosts of leishmaniasis.

## Introduction

Phlebotomine sand flies (Diptera: Psychodidae: Phlebotominae) are the biological vectors of a group of diseases that includes leishmaniasis, human bartonellosis, and sand fly fever [1, 2]. Leishmaniases are group of diseases caused by protozoan parasites of the genus *Leishmania* (order: Kinetoplastida; family: Trypanosomatidae) [3]. The diseases are range from self-healing cutaneous leishmaniasis (CL) to disfiguring diffuse cutaneous/post-kala-azar dermal leishmaniasis (DCL/PKDL) and the fatal visceral leishmaniasis (VL, kala-azar) [4]. The diseases are epidemiologically complex, involving multiple vector species and reservoir hosts, and diverse transmission cycles [5]. In Sudan, CL has been endemic since 1910 [6], caused by *Le*. *major* and transmitted by *Phlebotomus papatasi* [7]. CL was endemic in western parts of Sudan before 1970, but after a major epidemic along the River Nile, the disease became endemic in many regions of the country [7, 8]. While in Sudan, VL is caused by *Le*. *donovani* and transmitted by *Ph*. *orientalis*. The major VL endemic areas in Sudan are in the eastern, central, and southern regions; several other areas of sporadic occurrence are scattered in Kordofan states and in the central and western parts of Darfur states [9]. Transmission of the disease takes place both in *Acacia seyal* (Taleh) and *Balanites aegyptiaca* (Higleeg) woodland and inside villages [10]. It is probable that both anthroponotic and zoonotic transmission of *Le*. *donovani* takes place in eastern Sudan [3].

Sand fly vectors transmit several etiological agents through feeding on a wide variety of hosts, such as humans, livestock, dogs, and chickens [11]. The blood meal is essential for egg development and various physiological processes, and sand flies can acquire or transmit pathogens by this means [12]. Detailed knowledge of the preferred vertebrate hosts and feeding behavior of sand fly vectors is considered to be a prerequisite for a successful prevention and control program implementation and evaluation of changes in human-vector contact during intervention programs [13].

Blood meal analysis of hematophagous arthropods is considered a practical approach to identifying their preferred hosts under natural conditions [14]. The anthropophilic index (percentage feeding on humans) is a vital component of the vectorial capacity of disease vectors [15–17], and knowledge of animal hosts is also crucial in identifying reservoirs of vector-borne zoonotic or enzootic pathogens [16].

Relatively, limited studies are available regarding blood meal analysis and host preference of different sand fly vectors, despite the variety of available techniques that are available. Methods used for blood meal analysis on sand flies are mostly derived from those used for mosquitoes [13]. However, many factors limit the use of this approach; sand flies are minute insects compared to mosquitoes and ingest less blood volume (0.3–0.6 μl per blood meal) and (2–6 μl per blood meal) respectively [13, 18, 19], and this reduces the active time period which to determine the blood meal source (24–48 hours) post blood meal ingestion [11, 19–22]. These difficulties impose critical challenges from disease ecology perspective and epidemiological assessment of disease transmission [23].

The successful typing of blood meals of wild-caught sand flies requires at least the rapid collection of engorged sand flies after obtaining blood meals. The blood-fed sand flies or their blood meals must be preserved appropriately to avoid degeneration of blood meal, and determination of the optimum concentration of blood meal extracted DNA used in PCR analysis [14, 24].

Most studies of blood meal sources of arthropod and sand fly vectors rely mostly on serological techniques; precipitin test, counter immune-electrophoresis, latex agglutination test and enzyme-linked immunosorbent assay (ELISA) [13, 25–27]. Although serological techniques are informative, they lack sensitivity, and they are also time-consuming [11, 17].

PCR-based methods can be considered as a convenient alternative for the identification of blood meals rather than serological techniques [28]. They can also be used for species confirmation, detection of infection by different pathogens, and population genetic studies, all on an individual specimen [14]. Several PCR-based methods have been used to identify blood meal sources of sand flies including one that targets the prepronociceptin (PNOC) gene [29–31], FTA-based technology [11], PCR-RFLP [32, 33]. Besides, real time PCR assays [34], multiplex PCR [35], barcoding PCR [23, 36, 37], PCR reverse-line blotting (RLB) and ELISA assays [17, 27, 28, 38–40].

The cytochrome *b* gene of mitochondrial DNA is conserved in all animals, and has distinct characteristics for each species, making it an ideal choice for identifying blood meal sources [41]. Using of cytochrome *b* gene is a powerful method for identification, due to the high copy number of mitochondrial gene and sufficient genetic variation at the primary sequence level among vertebrate taxa for reliable identification [14, 37]. Multiplex PCR is convenient as it immediately identifies species DNA, offering speed and cost-effectiveness to blood meal identification [42].

This study aimed to identify blood meal sources and host preferences among sand fly vectors (*Ph*. *orientalis* and *Ph*. *papatasi*) in endemic areas of Sudan using the multiplex PCR method.

## Materials and methods

### Study area

**Fig 1:**
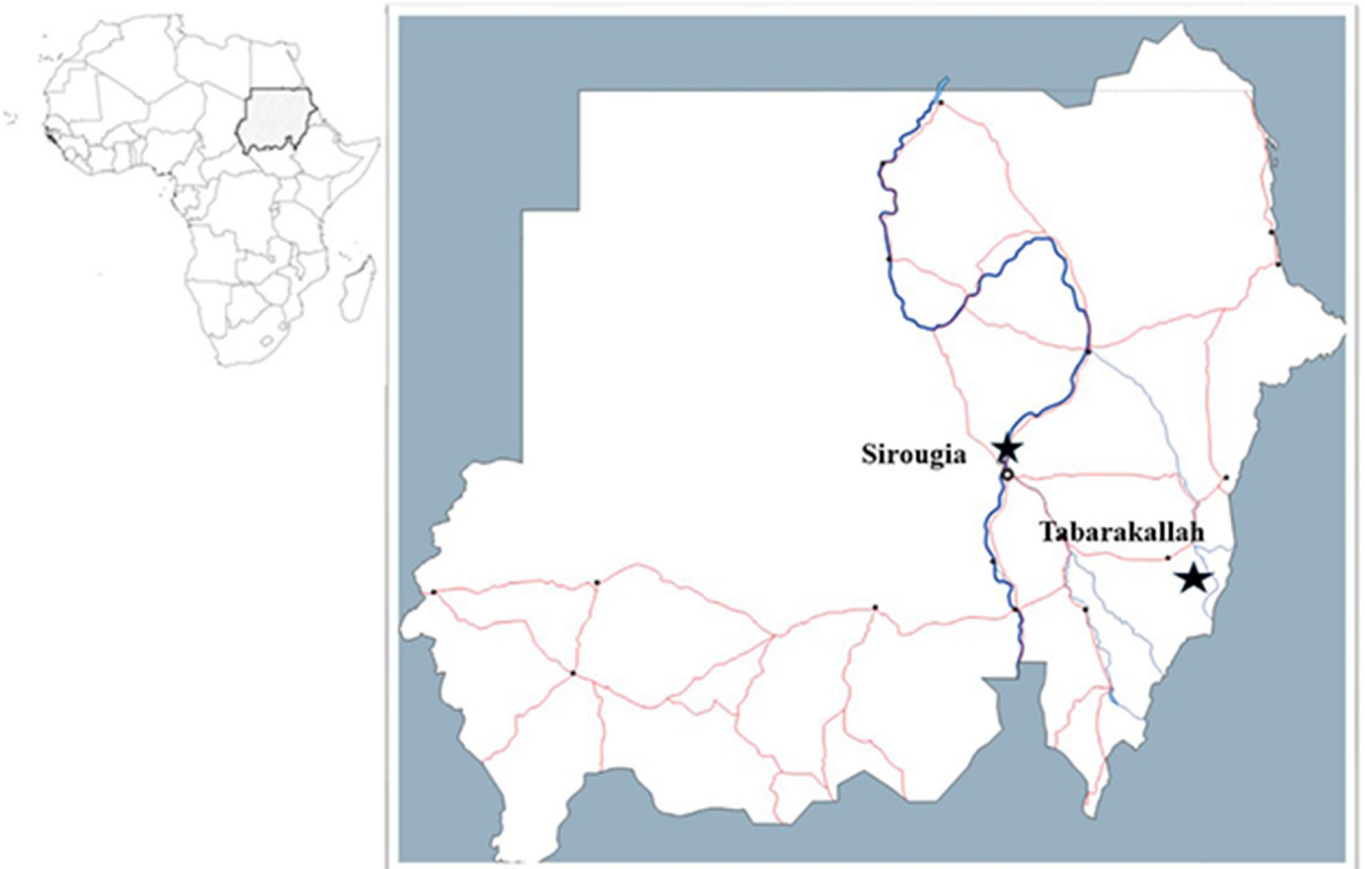
Regional and local map of the study sites in Sudan. The areas highlighted with black stars represent the study site villages: Sirougia in central Sudan and Tabarakallah in eastern Sudan.

Study samples were collected from two endemic sites of leishmaniasis with different ecologies; Sirougia village (15°45`N, 32°15`E) and Tabarakallah village (13° 37`N, 36° 05`E) (Fig 1). The Sirougia village is a green rich irrigated site with many agricultural schemes on the east bank of the River Nile, approximately 30 km north Khartoum state [7, 8]. It is located in a flat land covered by alluvium of silty clay and sand deposited by the River Nile. The climate is semi-desert with two distinct seasons, rainy season (July-October) and dry season (November-June). Vegetation in the area is composed of desert scrub trees such as *A*. *seyal* and *A*. *nilotica* (Sunt). The inhabitants of the village live in homes made of clay layers or bricks, roofed with grass and mud or corrugated iron sheets. Several previous CL outbreaks have occurred in the Sirougia village, whilst some of the uncommon CL cases have been reported in the surrounding communities [6, 7].

Tabarakallah village is a known endemic site of VL [43] that lies near the Atbara River. The soil is mainly chromic vertisol soil (black cotton soil), the climate is temperate savannah with a rainy season (May-September) and dry season (October-April). The dominant trees there are *A*. *seyal*, *B*. *aegyptiaca* and *A*. *senegal* (Hashab). The people of the village lived in a typical African hut constructed of wood, bamboo, and grass [44, 45].

### Sand fly collection

Sand flies were collected at night from homes and peri-domestic sites in the two villages using the CDC miniature light trap (Model 2836BQ, USA) and sticky traps [46]. Both traps were set up from 6:00 pm—6:00 am for five consecutive nights in the two villages, during March—April 2018 in accordance with the increased numbers of phlebotomine sand flies during dry season [3, 47]. The collected sand flies were sorted out, preserved in absolute ethanol and kept at -20°C for further analysis.

Ethical approval for this study was taken from Noguchi Memorial Institute for Medical Research-IRB, University of Ghana, Ghana (study no. 065/15-16) and informed consent was obtained from owners of homes in villages.

### Morphological identification of sand fly vectors

*Phlebotomus* (blood-fed and unfed) sand flies collected from the wild were sorted out from other *Sergentomyia* species and then mounted individually in Puri’s media on glass slides, as described by [48]. Using sterilized fine forceps and dissecting micro-needles, the head containing the pharynx and cibarium, and the last four terminal segments of the abdomen were separated under the dissecting microscope and covered with coverslips. The rest of the blood-fed specimen was placed individually in 1.5 ml Eppendorf tubes containing absolute ethanol and labelled with a specific code that denoted the number assigned to the dissected parts mounted on the glass slide, and then stored at -20 ºC for subsequent molecular investigation.

Morphological identification of the wild-caught phlebotomine (blood-fed and unfed) sand fly was done under a binocular microscope at 40X following the keys of [49–51]. The primary morphological features used for the female sand fly identifications were the spermatheca, the pharynx, and the cibarial armature.

### DNA extraction and molecular identification of sand fly vectors

The thorax and remaining part of the abdomen of each blood-fed female *Phlebotomus* specimen was used for genomic DNA extraction as described by [52], with some modifications. Briefly, tissues were first washed three times with distilled water to remove the traces of the ethanol. The washed samples were placed individually in 1.5μl Eppendorf tube and homogenized in 50μl grinding buffer (0.1mM NaCl, 0.1M Tris HCl pH 8.0) using a sterile glass pestle. The homogenates were incubated at 95°C for 30 min, kept at -20°C for 30 min and then centrifuged for 5 min at 14,000 rpm. The supernatants were transferred to fresh Eppendorf tubes, and 30μl of DNA-free water was added and stored at -20°C for further molecular investigations.

Species-specific primers, published by [53], were used for the identification of *Ph*. *(Phlebotomus)* ITS2 (rDNA) (Macrogen Inc. Korea); UN-F-JTS4: GCAGCTAACTGTGTGAAAT; UN-R-C1a (*Ph*. *papatasi*): CCTGGTTAGTTTCTTTTCCTCCGCT; PdubSSP (*Ph*. *duboscqi*): CCATAGCTCCAATGTTTTAC; PbergSSP (*Ph*. *bergeroti*): CGTGGTCCATGCAGTTTAAATG, yielding a PCR product of 500 bp (*Ph*. *papatasi*); 200 bp (*Ph*. *duboscqi*); 100 bp (*Ph*. *bergeroti*).

Amplification of the samples was done in a total of 25μl volume by adding 8μl DNA template, PCR water and 10pmol of each primer (UN-F-JTS4, UN-R-C1a, PdubSSP, and PbergSSP) to Maxime PCR premix kit (iNtRON Biotechnology Inc., South Korea) containing (5U/μl) i-Taq DNA polymerase, 2.5mM each of dNTPs, 10x reaction buffer, and 1x gel loading buffer. All PCR reactions were run on a thermocycler (SensoQuest, Germany). The PCR cycling program was as follows: an initial denaturation at 95°C for 2 min, 25 cycles of denaturation at 94°C for 30 second, annealing at 54°C for 30 second, extension at 72°C for 45 second and final extension at 72°C for 7 min. Each reaction included one negative PCR control (no DNA template) and positive controls of *Ph*. *papatasi*, *Ph*. *duboscqi*, and *Ph*. *bergeroti* DNA.

For *Ph*. *orientalis* samples, species-specific primers published by [54, 55] based on mtDNA Cytb were used; MM2F: TTTACTCTCTGCTATTCCTTATCTAGG and MM2R: TCTCAGATTTTTGAAATTAGAGGATTT (Macrogen Inc. Korea), yielding amplicon of 675 bp. Amplification of the samples was performed in a total of 25μl volume by adding 8μl DNA template, PCR water and 10pmol of each primer (MM2F and MM2R) to the Maxime PCR premix kit (iNtRON Biotechnology Inc., South Korea). The PCR cycling was modified as follows; an initial denaturation at 95°C for 2 min, 14 cycles of denaturation at 95°C for 30 second, annealing at 61.4°C decreased 0.5°C per cycle for 30 second, extension at 72°C for 70 second, 19 cycles of denaturation at 95°C for 30 second, annealing at 54.4°C for 30 second, extension at 72°C for 70 second and final extension at 72°C for 5 min. Each reaction included one negative PCR control and positive control of *Ph*. *orientalis* DNA.

Five μl of the PCR product was electrophoresed on a 1.5% agarose gel stained with Ethidium bromide [56]. A 100 bp DNA ladder was loaded for the determination of band size. The gel was run in 1x TBE buffer (Tris base, boric acid, EDTA pH 8.3) for one hour at 90V. The PCR products were observed under UV illumination and documented using the BDA gel image documentation system (Biometra Analytika Jena Company, Germany).

### Identification of blood meal source using multiplex PCR

A set of primers that amplify segments of cytochrome *b* gene of vertebrate mtDNA were used following [32, 35]; UNREV1025A “universal”: GGTTGTCCTCCAATTCATGTTA; UNFOR403 “mammals”: TGAGGACAAATATCATTCTGAGG; Human 741F: GGCTTACTTCTCTTCATTCTCTCCT; Goat 894F: CCTAATCTTAGTACTTGTACCCTTCCTC; Cow121F: CATCGGCACAAATTTAGTCG; Dog 368F: GGAATTGTACTATTATTCGCAACCAT (Macrogen Inc. Korea), yielding PCR products of 623bp, 334bp, 132bp, 561bp, and 680bp respectively. PCR amplification was performed in a total of 25μl volume that comprised of 8μl DNA template, PCR water, and 10pmol of each primer (UNFOR403 and UNREV1025A; UNREV1025A, Human 741F, Goat 894F, Cow121F, and Dog 368F) added to the Maxime PCR premix kit (iNtRON Biotechnology Inc., South Korea). PCR reaction was run on a thermocycler (SensoQuest, Germany). The multiplex PCR cycling condition was modified from previous publications [32, 35] as follows: an initial denaturation at 95°C for 5 min, 35 cycles of denaturation at 95°C for 60 seconds, annealing at 58°C for 60 seconds, extension at 72°C for 60 seconds, and final extension at 72°C for 7 min. To each amplification test, one negative PCR control and positive controls of human, goat, cow, and dog DNA were included. The PCR products electrophoresed on 1.5% agarose gel and analyzed as described above.

### Detection of *Leishmania* parasite in the blood-fed phlebotomine vectors

*Leishmania* DNA was detected among blood-fed sand fly vectors using the method of [57]. The primers 18S-LEISH forward: GCTGTGCAGGTTTGTTCCTG′3 and 18S-LEISH reverse: GGACGCACTAAACCCCTCAA (Macrogen Inc. Korea), were used to amplify DNA amplicon of 357bp within the 18S rRNA gene of *Le*. *donovani*. PCR was performed in 25μl reaction volume using Maxime PCR premix (iNtRON Biotechnology Inc., South Korea) according to the manufacturer’s instructions. Cycling conditions were done with minor modification from previous report [57]; an initial denaturation at 95°C for 2 min, 30 cycles of denaturation at 95°C for 30 second, annealing at 59.3°C for 30 seconds, extension at 72°C for 40 second, and a final extension at 72°C for 5 min. One negative PCR control and positive control of *Le*. *donovani* DNA were included in every PCR run. PCR products were electrophoresed on 1.5% agarose gel and analyzed as described above.

## Results

The total number of collected phlebotomine (blood-fed and unfed) sand flies from Sirougia was 50 females, while in Tabarakallah it was 130 females. The morphological identification revealed 103 (57.2%) *Ph*. *orientalis*, 42 (23.3%) *Ph*. *papatasi* and 31 (17.2%) *Ph*. *bergeroti* were found in both Sirougia and Tabarakallah sites, while *Ph*. *rodhaini* 2 (1.1%) and *Ph*. *duboscqi* 2 (1.1%) were found only in Tabarakallah site (Table 1). Details of abundance of each species per site are reported in Table 1.

**Table 1:** Numbers and percentages of female phlebotomine sand fly vectors collected from two study sites in Sudan during 2018.

Sand fly species	Sirougia village		Tabarakallah village		Total
	Blood-fed	Unfed	Blood-fed	Unfed	
***Phlebotomus orientalis***	0 (0%)	1 (2%)	15 (11.5%)	87 (66.9%)	103 (57.2%)
***Ph*. *rodhaini***	0 (0%)	0 (0%)	0 (0%)	2 (1.5%)	2 (1.1%)
***Ph*. *papatasi***	4 (8%)	21 (42%)	3 (2.3%)	14 (10.8%)	42 (23.3%)
***Ph*. *bergeroti***	5 (10%)	19 (38%)	4 (3.1%)	3 (2.3%)	31 (17.2%)
***Ph*. *Duboscqi***	0 (0%)	0 (0%)	0 (0%)	2 (1.5%)	2 (1.1%)
**Total**	9 (18%)	41(82%)	22 (16.9%)	108 (83.1%)	180

The total number of blood-fed phlebotomine females in both sites was 31 (17%) specimens. Out of the total blood-fed females, 9 (18%) were found on Sirougia site, while 22 (16.9%) were found on Tabarakallah site (Table 1).

Results of molecular identification of blood-fed specimens confirmed the presence of *Ph*. *bergeroti* (N = 5, 10%) and *Ph*. *papatasi* (N = 4, 8%) in Sirougia site, while in Tabarakallah, *Ph*. *orientalis* (N = 15, 11.5%) was confirmed in addition to *Ph*. *papatasi* (N = 3, 2.3%) and *Ph*. *bergeroti* (N = 4, 3.1%) (Table 1).

**Fig 2:**
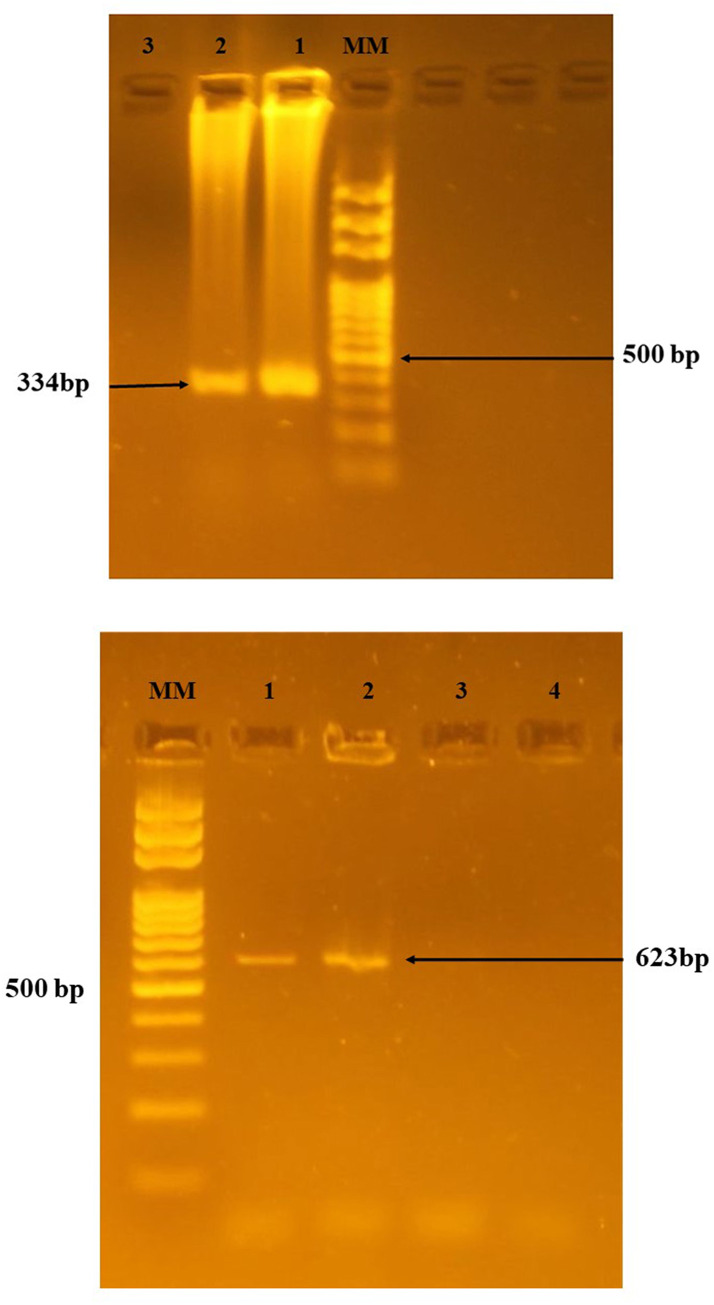
A. Electrophoresis of DNA multiplex polymerase chain reaction profile (1.5% agarose gel) after amplification of cytochrome *b* gene of vertebrate mtDNA fragments among blood-fed *Phlebotomus orientalis* using UNREV1025A, Human 741F, Goat 894F, Cow121F, and Dog 368F primers. MM: 100 bp DNA molecular marker; lane 1: human blood; lane 2: human blood; lane 3: negative control (PCR water). B. Electrophoresis of DNA multiplex polymerase chain reaction profile (1.5% agarose gel) after amplification of cytochrome *b* gene of mammalian mtDNA fragments among blood-fed *Phlebotomus orientalis* using UNFOR403 and UNREV1025A primers. MM: 100 bp DNA molecular marker; lane 1: mammalian blood; lane 2: mammalian blood; lanes 3, 4: negative control (PCR water).

The results of blood meal sources showed that 2 (7%) *Ph*. *orientalis* specimens from Tabarakallah site had fed on humans (Fig 2A), and the anthropophilic index was 13.3%, while the other vertebrate hosts have not been detected in the rest of the samples. The results of human DNA were confirmed by using the universal primers of the mammalian host (Fig 2B). No *Leishmania* parasite DNA was detected in all blood-fed specimens.

## Discussion

An improved understanding of the feeding behaviors of sand flies could contribute to new, more effective strategies for the control of sand fly populations. The present study of host preference of sand fly vectors using multiplex PCR is the first to be conducted in leishmaniasis endemic sites in Sudan. In this study, sand fly blood meals were examined by multiplex PCR using specific primers for cytochrome *b* gene, distinguishing between human, goat, cow, and dog. The multiplex PCR protocol described here, allowed identification of blood meal sources of many vertebrate species in one assay.

Most collected specimens in the current study were *Ph*. *orientalis* (57.2%; Table 1), and the majority were from Tabarakallah site, the well-known endemic site of VL in eastern Sudan [43]. *Ph*. *orientalis* is a confirmed biological vector of VL in Sudan, parts of Ethiopia and Kenya [3, 10, 27, 44, 47, 58–68]. Recent studies in nearby endemic villages of VL in Ethiopia found that *Ph*. *orientalis* was the predominant feeder [17, 27, 67–69].

The presence of *Ph*. *papatasi*, *Ph*. *duboscqi* and *Ph*. *bergeroti* in the study sites are of epidemiological interest. The three species exist sympatrically in many places in central and eastern Sudan [7, 53, 70–72]. *Ph*. *papatasi* is a known vector transmitting CL in different parts of the country [6], while *Ph*. *duboscqi* and *Ph*. *bergeroti* have been involved in the transmission of *Le*. *major* in places, such as Ethiopia [73], Kenya [74], and Sahara [75].

Few specimens were identified as *Ph*. *rodhaini* (1.1%; Table 1). Generally, *Ph*. *rodhaini* is considered a rare species, some studies from Sudan reported small numbers of *Ph*. *rodhaini* captured from endemic sites [66, 68, 72, 76, 77] indicating its involvement in maintaining the transmission of VL between wild animals and dogs [66].

Hundreds of *Sergentomyia* sand flies were collected during the study period, and has been excluded from the study, because the genus *Sergentomyia* is known vector of the subgenus *Sauroleishmania* that infects reptiles and edentates [78–80] but not a proven biological vector of human leishmaniasis [79, 81]. However, some studies have reported the detection of different *Leishmania* species DNA in species of *Sergentomyia* [82–88]. It may be worth examining the biological role of this species in Sudan.

The results showed that 7% of wild-caught *Ph*. *orientalis* had fed on a human host in the VL community (Table 1), corroborating the role of this species as a proven vector of leishmaniasis in Sudan and eastern Africa [1, 2, 17]. The host preferences of *Ph*. *orientalis* collected from an area nearby Tabarakallah village with a similar environment revealed that 8.3% of meals from a human host [17], other studies showed that *Ph*. *orientalis* prefer feeding on humans and bovines [17, 27, 64, 67, 68, 69], these findings supported the fact that *Ph*. *orientalis* is a vector of VL in the area.

*Ph*. *orientalis* feed on wide range of hosts, this is remarkably affecting disease transmission, not only by offering alternative reservoir for the *Leishmania* parasites, but also by supporting survival of sand fly population through providing a continuous access to blood meal [64]. Reports from Sudan and New World showed that high sero-prevalence of *Le*. *donovani* and *Le*. *braziliensis* were found in donkeys, respectively [89, 90]. Also, dogs have been shown to have high sero-positivity for *Le*. *donovani* in Sudan and Ethiopia [91–95], and owning dogs is considered a risk factor for VL [96–98].

The identification of blood meal source of sand fly vectors is a challenge, as observed in this study, however, only two blood meal sources were successfully analyzed, while the rest failed, since the positive controls used during the tests showed the respective bands for each host. The justification for this failure could be attributed to the degraded and insufficient amounts of quality DNA from the tiny sand flies [14, 19], time of collection in response to blood digestion (more than 48 hrs) [22], and due to the limited set of primers which were used, implying blood sources other the studied animals.

No *Leishmania* DNA was detected among the screened low number samples, this result is not surprising, and is consistent with recent study done in northwestern Ethiopia [17], where the expected infection rate among phlebotomine vectors was 1–5 out of 1,000 flies [17, 44, 67]. PCR-based methods have been used previously for the detection of *Leishmania* in phlebotomine sand flies [17, 38, 99–102]. DNA prepared from the whole body of *Phlebotomus* sand flies not only can be used for blood meal identification, but can also be used for parasite detection by PCR simultaneously [11]. This simple method could be advantageous in epidemiological studies for parasite species identification in sand fly vectors. Also, it could be helpful in epidemic scenarios when and where robust, reliable results are urgently needed.

The correct identification of vertebrate hosts of sand flies is vital because it might reveal the nature of host preference and additional pathogen reservoirs to consider in the disease control. This knowledge can help in developing an effective disease control strategy that is considering patterns for the potential spread of vector-borne disease within and among communities. The details of sand fly blood-feeding patterns can also be used as a surveillance tool that assesses the efficacy and effectiveness of various prevention and control interventions, and to estimate the degree of coverage required for various leishmaniasis vaccines when developed [14].

Further studies investigating the host preference and feeding patterns of phlebotomine sand fly vectors should directly measure species composition of larger blood-fed sample size collected among dry and wet seasons from different leishmaniasis communities in Sudan to understand their vectorial capacity and to clarify natural transmission cycles.

## Conclusions

The blood meal identification in wild-caught sand flies can confirm a strong association between sand flies and reservoir hosts; in this case, humans in rural areas and help to improve our understanding of the role of reservoir host in the disease transmission. The rapid and higher sensitivity of the multiplex PCR would warrant its use in vector incrimination and reservoir determination of vector-borne diseases.

Standardization of blood meal study protocols, including the incorporation of vertebrate surveys and collection of sand fly vectors during dry and wet seasons, would improve prediction of the impact of vector-host interactions on disease ecology. The present study used analysis could be used to explore sand fly-vertebrate host association, in which host availability data are unavailable.

## Supporting information

S1 Raw image (PDF)

## List of abbreviations

Ph: *Phlebotomus*.
Le: *Leishmania*.
PCR: Polymerase chain reaction
DNA: Deoxyribonucleic acid
ELISA: Enzyme-linked immunosorbent assay
PNOC: Prepronociceptin
RFLP: Restriction fragment length polymorphism
RLB: Reverse-line blotting
VL: Visceral leishmaniasis
CL: Cutaneous leishmaniasis
ITS2: Internal transcribed spacer 2
rDNA: Ribosomal DNA
bp: Base pair.
mtDNA: Mitochondrial DNA
Cytb: Cytochrome b
TBE: Tris base EDTA
