# Supplementary material for: Studies of host preferences of wild-caught Phlebotomus orientalis and Ph. papatasi vectors of leishmaniasis in Sudan
Source: PLoS One. 2020 Jul 21;15(7):e0236253. doi: 10.1371/journal.pone.0236253 (PMC7373290; doi:10.1371/journal.pone.0236253)
Supplement: S1 Raw image — (PDF) [file pone.0236253.s001.pdf]

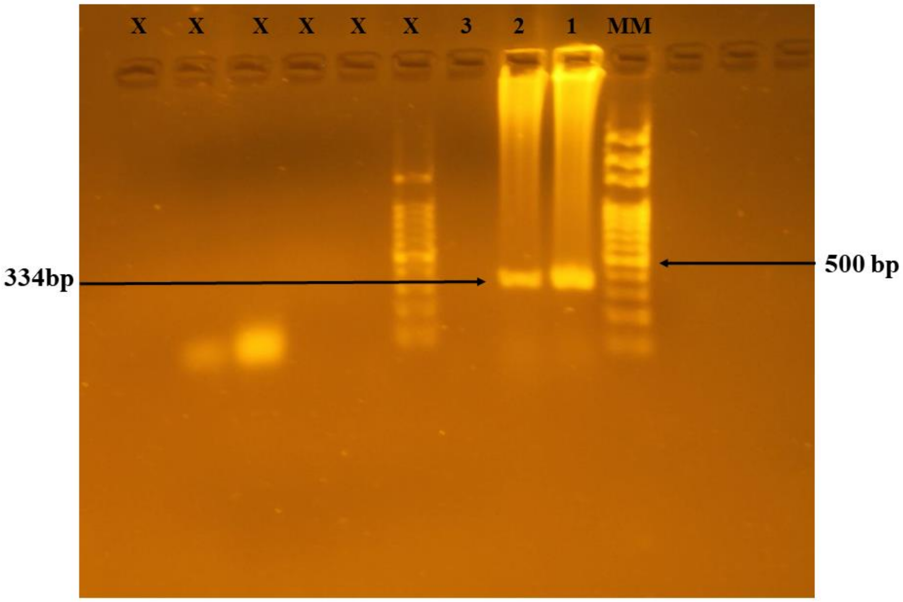

Electrophoresis of DNA multiplex polymerase chain reaction profile (1.5% agarose gel) after amplification of cytochrome *b* gene of vertebrate mtDNA fragments among blood-fed *Phlebotomus orientalis* using UNREV1025A, Human 741F, Goat 894F, Cow121F, and Dog 368F primers. MM: 100 bp DNA molecular marker; lane 1: human blood; lane 2: human blood; lane 3: negative control (PCR water).

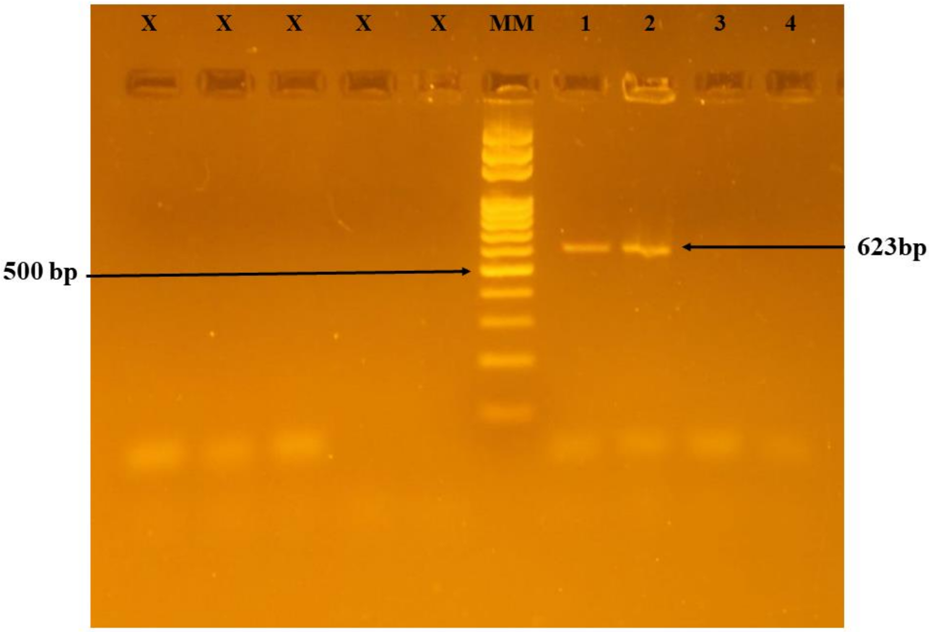

Electrophoresis of DNA multiplex polymerase chain reaction profile (1.5% agarose gel) after amplification of cytochrome *b* gene of mammalian mtDNA fragments among blood-fed *Phlebotomus orientalis* using UNFOR403 and UNREV1025A primers. MM: 100 bp DNA molecular marker; lane 1: mammalian blood; lane 2: mammalian blood; lanes 3, 4: negative control (PCR water).
